# Impact of Mirabegron Administration on the Blood Pressure and Pulse Rate in Patients with Overactive Bladder

**DOI:** 10.3390/medicina58060825

**Published:** 2022-06-19

**Authors:** Hidenori Ito, Tomohiro Matsuo, Kensuke Mitsunari, Kojiro Ohba, Yasuyoshi Miyata

**Affiliations:** Department of Urology, Nagasaki University Graduate School of Biomedical Sciences, Nagasaki 852-8501, Japan; ito218@nagasaki-u.ac.jp (H.I.); kmitsunari@nagasaki-u.ac.jp (K.M.); ohba-k@nagasaki-u.ac.jp (K.O.); yasu-myt@nagasaki-u.ac.jp (Y.M.)

**Keywords:** blood pressure, pulse rate, mirabegron, overactive bladder

## Abstract

*Background and Objectives:* To determine changes in the blood pressure (BP) and pulse rate (PR) before and after the administration of mirabegron in real-world clinical practice for patients with overactive bladder (OAB). *Materials and Methods:* This study was conducted in patients newly diagnosed with OAB. Before and 12 weeks after mirabegron treatment, we evaluated the effects on BP and PR. An overall examination was conducted, and the patients were divided into two groups according to their age: a young group (<65 years old) and an old group (≥65 years old). *Results:* A total of 263 patients were enrolled in this study. In the overall and intragroup comparisons, the systolic BP (SBP) did not change significantly after mirabegron administration. However, an increase in SBP of ≥10 mmHg was observed in 53 (20.2%), 4 (7.4%), and 49 (23.4%) in the entire group, young group, and old group, respectively (*p* = 0.009). Regarding diastolic BP, a significant decrease after the treatment was detected in entire (71.2 ± 11.4 versus 69.8 ± 10.7 mmHg; *p* = 0.041) and old patients (71.5 ± 10.6 versus 69.5 ± 10.2 mmHg; *p* = 0.012). There was no significant change in PR in our study population. Further examination using a propensity match score revealed that age was the risk factor for the increase in SBP after mirabegron administration. *Conclusions:* Mirabegron does not have any adverse effects on BP and PR. However, since some patients in this study had elevated SBP after administration, we suggest regular BP monitoring during mirabegron treatment.

## 1. Introduction

Overactive bladder (OAB) is a syndrome characterized by urinary urgency with or without urgency incontinence [1]. The prevalence of OAB increases with age; data from some epidemiological surveys suggest that the prevalence of OAB is between 16% and 19% [2,3,4]. Although behavioral therapy is the basis of treatment for OAB, various guidelines recommend anticholinergic agents and β3 adrenergic receptor agonists following behavioral therapy [5,6]. Although they are effective for OAB, many researchers have reported adverse events with anticholinergic drugs, including constipation and dry mouth [7]. In addition, there are concerns about the effects of β3 adrenoreceptor agonists on the circulatory system, such as hypertension and tachycardia [8]. However, the actual state of increase in blood pressure (BP) and pulse rate (PR) due to treatment with β3 adrenoreceptor agonists in real-world clinical practice is unclear.

OAB is more common in the elderly [9]. Aging is a major risk factor for hypertension and tachycardia, which are often associated with reduced quality of life and shortened life expectancy [10,11]. Based on these facts, in this study, we focused on the changes in BP and PR before and after administration of the β3 adrenoreceptor agonist mirabegron in patients with OAB. In addition, we investigated the influence of mirabegron on BP and PR separately in elderly patients (≥65 years old) and young patients (<65 years old).

Furthermore, we aimed to clarify the details of clinically significant risk factors for increased BP by oral mirabegron therapy in the real-world setting, including elderly patients with OAB.

## 2. Materials and Methods

### 2.1. Patients and Study Design

The study was approved by the appropriate ethics review board of Nagasaki University Hospital, Nagasaki, Japan (Registration No. 11120267) and was conducted in accordance with the principles of the Declaration of Helsinki. We conducted this prospective clinical study in a single center between September 2015 and March 2017 and included patients who were newly diagnosed with OAB. Based on the overactive bladder symptom score (OABSS), we defined participants with a urinary urgency (Question [Q]3) score of ≥2 and those with a total score of ≥3 as having OAB (Table 1) [12]. The exclusion criteria were as follows: (i) systolic BP (SBP) of 180 mmHg or more diastolic BP (DBP) of 110 mmHg with or without hypertension treatment, before OAB treatment; (ii) resting PR of ≤50/min or ≥110/min; (iii) serious heart disease; (iv) pregnancy or planned pregnancy; and (v) severe renal/hepatic dysfunction.

Patients with OAB received oral monotherapy with mirabegron (Betanis^®^; Astellas Pharma, Tokyo, Japan) 50 mg once daily after a meal for 12 weeks. The patients did not undergo combination therapy with mirabegron and anticholinergic drugs during the treatment period. Changes in OABSS during the study period were used to evaluate subjective symptoms. BP and PR were measured at the beginning of treatment and at 12 weeks after the study period. Antihypertensive agents and medication for benign prostatic hyperplasia, including α1 receptor antagonists and phosphodiesterase 5 inhibitors, were continued on the condition that the dose would be kept constant during the study period. We divided the patients into two groups by age: the young group (<65 years old) and the elderly group (≥65 years old). The significance of the differences between the two groups was evaluated. In addition, we investigated the proportion of patients with an increase in SBP of 10 mmHg or more, which is considered to be clinically significant [13] after taking mirabegron, and analyzed the details of risk factors for increased SBP using statistical methods.

### 2.2. Evaluation of BP and PR

We evaluated patient BP and PR using an automated oscillometric upper-arm BP monitoring device (HBP-9020, Omron Co., Kyoto, Japan). The participants were seated in a quiet room at a comfortable temperature (22–25 °C) and were instructed to avoid talking during the procedure. In this study, we evaluated the BP and PR of patients in a stable situation without a desire to urinate after visiting our hospital. The BP measurements were started after the participants had rested for 5 min. The participants sat on a chair with their legs uncrossed and their feet flat on the floor. All BP measurements were performed on the participant’s left arm at the level of the heart. The data were measured twice at intervals of approximately 2 min, and the average value was used as the measured value.

### 2.3. Evaluation of Objective Findings

Uroflowmetry and post-void residual urine volume (PVR) were used to assess the objective symptoms. The maximum flow rate (Qmax) was measured using the Duet^®^ Logic G2 system (Mediwatch UK Ltd., Rugby, UK) on free uroflowmetry and PVR using transabdominal ultrasound sonography (HI VISION Avius^®^, Hitachi-Aloka Medical, Ltd., Tokyo, Japan).

### 2.4. Propensity Score Matching

The elderly patients with OAB were matched (1:1 ratio) with young patients with OAB according to their propensity score through nearest-neighbor matching based on their characteristics. We set the caliper width to 0.2 SDs.

### 2.5. Statistical Analyses

All data are presented as mean ± SD. The paired *t*-test, Wilcoxon signed-rank test, Student’s *t*-test, and Mann–Whitney U test were used to evaluate changes in subjective and objective symptoms as required. All tests were two-sided, and the statistical significance was set at *p* < 0.05. All statistical analyses were performed using the JMP 15 software (SAS Institute, Cary, NC, USA). The number of samples was determined on the basis of previous reports [14,15,16]. Hence, we set a probability of 0.05 (two-sided), a power of 80%, and an effect size of 0.5. We estimated that the ideal number of participants for this study should be at least 239.

## 3. Results

### 3.1. Patients’ Characteristics

**Figure 1:**
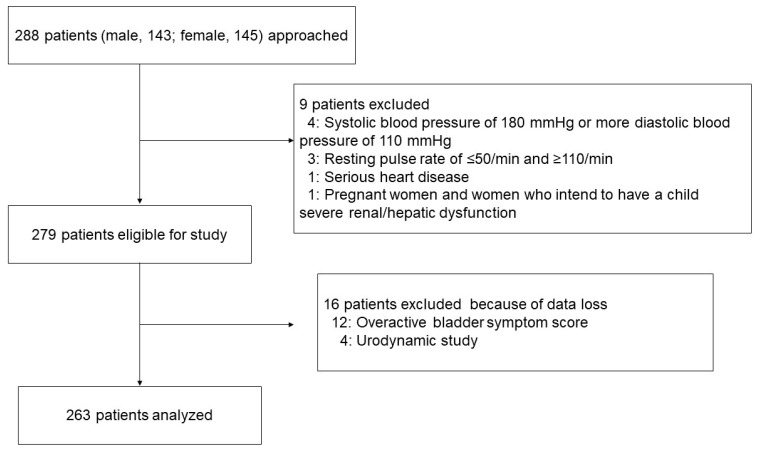
Patients flow diagram.

A total of 263 patients, comprising 131 men and 132 women who fulfilled the criteria for OAB, were enrolled in the study (Figure 1). As shown in Table 2, the mean age was 73.1 ± 11.6 years. The mean SBP and DBP before treatment were 127.5 ± 16.2 mmHg and 71.2 ± 11.4 mmHg, respectively, and the mean PR was 74.5 ± 12.1/min. Prior to this study, 128 patients (48.7%) had hypertension. All hypertensive patients were prescribed antihypertensive drugs. There was no significant difference between the two groups in the proportion of the types of antihypertensive agents used (*p* = 0.912) (Table 2). In addition to age, the proportion of men in the old group was significantly higher than in the young group (*p* < 0.001). Benign prostatic hyperplasia was diagnosed before the start of this study in 94 (71.8%) male patients with OAB, although there was no significant difference in treatment between the two groups (Table 2).

Regarding subjective symptoms, OABSS Q2 (nocturia), Q3, and the total score was significantly higher in the elderly group (Q2, *p* = 0.007; Q3, *p* = 0.010; total score, *p* = 0.011). Voided volume and Qmax were significantly lower in the elderly group than in the young group (voided volume, *p* < 0.001; Qmax, *p* < 0.001). The proportion of patients with hypertension, hyperlipidemia, and chronic renal dysfunction was higher in the elderly group (hypertension, *p* < 0.001; hyperlipidemia, *p* = 0.023; chronic renal dysfunction, *p* = 0.020) (Table 2).

### 3.2. Efficacy of Mirabegron for OAB Symptoms

Table 3 shows the efficacy of mirabegron for OAB symptoms. All items of the OABSS and its total score in the total group and the elderly group improved after mirabegron administration. Conversely, in the young group, OABSS Q2, Q3, and total scores improved after mirabegron administration. Voided volume significantly improved after treatment with urodynamic parameters in both groups. However, the Qmax and PVR did not change before and after mirabegron administration (Table 3).

### 3.3. Changes in the Hemodynamics Status during Mirabegron Treatment

The SBP at 12 weeks after the administration of mirabegron was 126.2 ± 14.9 mmHg; there was no significant difference from the SBP of 127.5 ± 16.2 mmHg before administration (*p* = 0.148). The DBP was 71.2 ± 11.4 mmHg before administration and 69.8 ± 10.7 mmHg at 12 weeks after treatment, showing a significant decrease (*p* = 0.041) (Table 4). Intragroup comparison between the young and elderly groups found that there were no significant changes in either SBP or DBP before and after administration of mirabegron in the young group. In the elderly group, there was no change in SBP before and after mirabegron administration; however, the DBP was 71.5 ± 10.6 mmHg before treatment and 69.5 ± 10.5 mmHg after 12 weeks of treatment (*p* = 0.012, Table 4).

The resting pulse, which was 74.5 ± 12.1/min before administration, was 74.0 ± 11.5/min after 12 weeks (*p* = 0.449). By age group, the PR in the young group changed from 75.0 ± 11.0/min to 74.9 ± 12.2/min (*p* = 0.871), and that in the elderly group changed from 74.3 ± 12.3/min to 73.8 ± 11.3/min (*p* = 0.453). However, no significant changes in PR were observed in either group (Table 4).

An increase in SBP of ≥10 mmHg was observed in 53 (20.2%) patients in the total study population; 4 (7.4%) patients in the young group and 49 (23.4%) patients in the elderly group (*p* = 0.009).

When the patients were divided into two groups according to the presence or absence of hypertension, there were no significant differences in SBP and DBP in either group before and after mirabegron administration. Similarly, there was no significant difference in PR before and after mirabegron treatment in either group (Table 5). Furthermore, none of the antihypertensive agents administered orally affected the fluctuations in BP and PR caused by mirabegron (Table 6).

### 3.4. Predictive Marker for the Clinically Significant Elevation of SBP

We used logistic regression analysis to identify risk factors for clinically significant elevations in SBP. Only age (≥65 years), which was the cut-off for the definition of the elderly group in this study, was a risk factor for increased SBP in the univariate analysis (Table 7). Based on the above results, we evaluated whether age is a risk factor for increased SBP after administration of mirabegron using propensity score matching (Table 8 and Table 9). The study analyzed the data of 102 patients (51 patients in each group cohort). The standardized mean difference of all characteristics was < 0.1, indicating negligible baseline differences between the groups. The incidence of SBP elevation (*p* = 0.002; 95% confidence interval, 1.99–20.70; odds ratio, 6.410) was significantly higher in the elderly group than in the young group (Table 9).

### 3.5. Adverse Events

A total of 3 patients (1.1%) reported mild dysuria, and 3 (1.1%) reported gastrointestinal symptoms such as heartburn and stomach upset. However, all adverse events were mild, and all patients continued oral mirabegron treatment for a 12-week study period.

## 4. Discussion

This clinical study showed that 12-week mirabegron oral administration is effective for OAB symptoms and is sufficiently safe in terms of the hemodynamic changes in BP and PR. In addition, intragroup comparisons by age showed no increase in BP or PR in either the young or elderly group.

Anticholinergic drugs are recognized as the standard treatment for OAB and are widely used. However, in some patients, anticholinergic drugs induce severe adverse events such as constipation, dry mouth, and cognitive dysfunction [7]. In addition, an excessive anticholinergic drug burden not only impairs the quality of life of patients due to adverse events but may also affect prognosis, particularly in elderly patients [17]. Hence, in clinical practice, treatment with safer drugs is desirable.

Large-scale clinical studies have reported that β3 adrenergic receptor stimulants, which are novel agents for OAB, are effective and relatively safe [18,19,20]. Mirabegron is a specific agonist that acts on β3-adrenoceptors in the detrusor muscle, the stimulation of which leads to active relaxation of the detrusor muscle in the storage phase, which increases bladder capacity without exerting an effect on voiding [21]. Similarly, in this study, 12-week oral mirabegron therapy for newly diagnosed patients with OAB significantly improved both subjective symptoms and objective parameters without deteriorating voiding symptoms. Recent large-scale and meta-analysis studies have reported that mirabegron does not have a significant effect on BP and PR [16,22]. However, a widely disseminated letter from direct healthcare professionals indicated that mirabegron is contraindicated in patients with severe uncontrolled hypertension (SBP ≥ 180 mmHg and/or DBP ≥ 110 mmHg). Therefore, in the present study, we focused on the changes in BP and PR in the real-world setting based on the analysis by age, which is one of the representative risk factors of hypertension.

In this study, we did not find a significant increase in SBP after 12-week oral mirabegron administration in the total participants group. Generally, elderly people are more likely to have hypertension; however, Hoffman et al. reported that mirabegron could be safely used even in elderly patients with OAB without affecting their cardiovascular risk, including BP [23]. In this study, none of the patients with OAB had an elevation of SBP above 180 mmHg after mirabegron administration, and across all the groups, there were no significant changes in SBP.

However, in a meta-analysis of patients with hypertension aged 60 years or older, when the increase in SBP exceeded 10 mmHg, a deterioration in total mortality and an increase in stroke occurrence were observed [13]. In this regard, our results showed that 20.2% of patients, irrespective of age, had a clinically significant increase in SBP of 10 mmHg or higher after oral mirabegron treatment. In addition, as revealed in this study, we showed for the first time that age ≥ 65 years can be a risk factor for the elevation of SBP after mirabegron administration using propensity score matching analysis. We consider this to be an important finding and propose regular monitoring of BP changes in elderly patients during treatment with mirabegron, even in those with stable BP prior to the commencement of treatment.

Conversely, DBP elevation was not observed during the 12-week study period. A previous study targeting men also did not find an elevation in the DBP [24]. In addition, Katoh et al. reported a slight decrease in DBP after a 12-week mirabegron add-on therapy to tamsulosin [22]. In the present study, DBP also decreased in the elderly group after oral mirabegron therapy. We postulate that this finding may be explained as follows. Aging causes an increase in oxidative stress and is a risk factor for hypertension and other hemodynamic changes [25]. A basic study using animal models reported that β3 adrenergic receptor agonists increased endothelial nitric oxide synthase (eNOs) activity and suppressed the development of superoxide [26]. This may indicate that, in some patients, particularly the elderly, administration of mirabegron results in increased nitric oxide production and reduced DBP by promoting vasodilation, as found in the present study. However, there is currently insufficient evidence to explain the results of the present study using this hypothesis, and further studies are necessary. In addition, in this study, DBP decreased in the group of patients who underwent treatment with drugs other than angiotensin II receptor blockers monotherapy for hypertension, although this difference was not significant. Further research is required to investigate the effects of the combination of antihypertensive agents and β3 agonists on hemodynamics.

The PR did not change significantly during the 12-week study period. Previous investigations have indicated that mirabegron may marginally increase heart rate [27,28], although the clinical relevance of this change is unknown. In addition, it has been reported that administration of mirabegron caused an increase in the PR of approximately 1.2/min, and that this increase was particularly remarkable in elderly patients aged 75 years and older [22]. However, another study reported that mirabegron therapy did not affect PR [29]. Our study supported these findings amongst the elderly and showed no increase in PR in either young or elderly patients. Further research is necessary to corroborate these findings.

There are some limitations to the present study other than those mentioned above. First, the number of samples was relatively small, and cardiac function could not be evaluated using electrocardiography or echocardiography. In addition, we did not have complete details of the patients’ medication status at the start of this study, except for information on antihypertensive agents. As mentioned above, patients with uncontrolled cardiovascular, renal, and hepatic diseases were excluded to avoid bias. We consider that our results will be useful in clinical practice when considering hemodynamic stability for patients with OAB. However, our results cannot be generalized to patients with untreated systematic diseases, including cardiovascular disorders.

## 5. Conclusions

This study found that mirabegron could be safely used with minimal fluctuations in BP and PR. However, as elevated BP was observed in some patients, we suggest regular BP monitoring during treatment with mirabegron, particularly for elderly patients.

## Figures and Tables

**Table 1:** Overactive bladder symptom score [12].

Question	Frequency	Score
1. How many times do you typically urinate from waking in the morning until sleeping at night?	≤7	0
	8–14	1
	≥15	2
2. How many times do you typically wake up to urinate from sleeping at night until waking in the morning?	0	0
	1	1
	2	2
	≥3	3
3. How often do you have a sudden desire to urinate, which is difficult to defer?	Not at all	0
	Less than once a week	1
	Once a week or more	2
	About once a day	3
	2–4 times a day	4
	5 times a day or more	5
4. How often do you leak urine because you cannot defer the sudden desire to urinate?	Not at all	0
	Less than once a week	1
	Once a week or more	2
	About once a day	3
	2–4 times a day	4
	5 times a day or more	5

**Table 2:** Patients’ characteristics.

	Total Group	Young Group	Elderly Group	*p*
Number of patients (Male, %)	263 (131, 49.8)	54 (15, 27.8)	209 (116, 55.5)	<0.001
Age (years)	73.1 ± 11.6	54.9 ± 7.7	77.8 ± 6.8	<0.001
Body mass index (kg/m^2^)	22.6 ± 3.9	23.2 ± 5.1	22.5 ± 3.6	0.938
Overactive bladder symptom score				
Q1 Daytime frequency	1.0 ± 0.7	0.9 ± 0.7	1.0 ± 0.7	0.689
Q2 Nighttime frequency	2.3 ± 0.7	2.1 ± 0.7	2.4 ± 0.7	0.007
Q3 Urgency	3.1 ± 1.3	2.9 ± 1.3	3.1 ± 1.2	0.010
Q4 Urgency incontinence	1.1 ± 1.3	0.9 ± 1.3	1.1 ± 1.4	0.225
Total score	7.5 ± 2.7	6.9 ± 2.5	7.6 ± 2.7	0.011
Urodynamic study				
Voided volume (mL)	154.2 ± 52.8	178.1 ± 61.4	148.1 ± 48.6	<0.001
Qmax (mL/sec)	14.6 ± 5.0	17.4 ± 4.6	13.9 ± 4.8	<0.001
Post-void residual urine (mL)	24.2 ± 12.3	23.0 ± 14.6	24.5 ± 11.7	0.167
Blood pressure				
Systolic blood pressure (mmHg)	127.5 ± 16.2	124.2 ± 15.0	128.4 ± 16.4	0.148
Diastolic blood pressure (mmHg)	71.2 ± 11.4	70.3 ± 14.0	71.5 ± 10.6	0.390
Pulse rate (/min)	74.5 ± 12.1	75.0 ± 11.0	74.3 ± 12.3	0.677
Comorbidity				
Hypertension (%)	128 (48.7)	12 (22.2)	116 (55.5)	<0.001
Diabetes Miletus (%)	29 (11.0)	6 (11.1)	23 (11.0)	1.000
Hyperlipidemia (%)	41 (15.6)	3 (5.6)	38 (18.2)	0.023
Chronic renal dysfunction (%)	78 (29.7)	6 (16.7)	69 (33.0)	0.020
Treatment for Hypertension				0.912
ARBs (% in Hypertension)	46 (35.9)	4 (8.7)	42 (91.3)	
CCBs (% in Hypertension)	33 (25.8)	4 (12.1)	29 (87.9)	
ARBs + CCBs (% in Hypertension)	28 (21.9)	2 (7.1)	26 (93.9)	
ARB + diuretics (% in Hypertension)	16 (12.5)	2 (12.5)	14 (87.5)	
Diuretics (% in Hypertension)	5 (3.9)	0 (0)	5 (100)	
BPH (% in male)	94 (71.8)	9 (60.0)	85 (73.3)	0.360
Treatment for BPH (% in BPH)	93 (98.9)	8 (88.9)	85 (100)	0.096
α1 receptor antagonists (% in BPH)	87 (93.5)	7 (87.5)	80 (94.1)	0.426
Phosphodiesterase 5 inhibitors (% in BPH)	6 (6.5)	1 (12.5)	5 (5.9)	

ARBs, angiotensin II receptor blockers; CCBs, calcium channel blockers; BPH, benign prostatic hyperplasia.

**Table 3:** Efficacy of mirabegron for the subjective and objective symptoms.

	Total Group			Young Group			Elderly Group		
	0 W	12 W	*p*	0 W	12 W	*p*	0 W	12 W	*p*
OABSS									
Q1 Daytime frequency	1.0 ± 0.7	0.8 ± 0.7	<0.001	0.9 ± 0.7	0.9 ± 0.6	0.833	1.0 ± 0.7	0.8 ± 0.7	<0.001
Q2 Nighttime frequency	2.3 ± 0.7	1.8 ± 0.8	<0.001	2.1 ± 0.7	1.6 ± 0.8	<0.001	2.4 ± 0.7	1.8 ± 0.8	<0.001
Q3 Urgency	3.1 ± 1.3	1.2 ± 1.1	<0.001	2.9 ± 1.3	1.4 ± 1.3	<0.001	3.1 ± 1.2	1.2 ± 1.1	<0.001
Q4 Urgency incontinence	1.1 ± 1.3	0.7 ± 0.9	<0.001	0.9 ± 1.3	0.8 ± 1.0	0.324	1.1 ± 1.4	0.7 ± 0.9	<0.001
Total score	7.5 ± 2.7	4.5 ± 2.5	<0.001	6.9 ± 2.5	4.7 ± 2.7	<0.001	7.6 ± 2.7	4.4 ± 2.5	<0.001
Urodynamic study									
VV (mL)	154.2 ± 52.8	179.8 ± 54.2	<0.001	178.1 ± 61.4	203.8 ± 67.9	0.005	148.1 ± 48.6	173.6 ± 48.4	<0.001
Qmax (mL/s)	14.6 ± 5.0	13.6 ± 4.5	0.228	17.4 ± 4.6	16.7 ± 3.9	0.119	13.9 ± 4.8	13.3 ± 4.4	0.201
PVR (mL)	24.2 ± 12.3	25.1 ± 12.4	0.120	23.0 ± 14.6	23.6 ± 12.9	0.230	24.5 ± 11.7	25.8 ± 12.2	0.315

OABSS, overactive bladder symptom score; VV, voided volume; Qmax, maximum flow rate; PVR, post-void residual urine.

**Table 4:** Changes in the blood pressure and pulse rate.

	Total Group			Young Group			Elderly Group		
	0 W	12 W	*p*	0 W	12 W	*p*	0 W	12 W	*p*
SBP(mmHg)	127.5 ± 16.2	126.2 ± 14.9	0.148	124.2 ± 15.0	122.3 ± 16.0	0.282	128.4 ± 16.4	127.2 ± 14.4	0.266
DBP(mmHg)	71.2 ± 11.4	69.8 ± 10.7	0.041	70.3 ± 14.0	71.0 ± 12.5	0.681	71.5 ± 10.6	69.5 ± 10.2	0.012
Pulse rate (/min)	74.5 ± 12.1	74.0 ± 11.5	0.449	75.0 ± 11.0	74.9 ± 12.2	0.871	74.3 ± 12.3	73.8 ± 11.3	0.453

SBP, systolic blood pressure; DBP, diastolic blood pressure.

**Table 5:** Changes in the hemodynamics status with and without hypertension.

	Normotensive *n* = 135			Hypertensive *n* = 128		
	0 W	12 W	*p*	0 W	12 W	*p*
SBP(mmHg)	123.1 ± 16.3	122.1 ± 14.5	0.916	132.3 ± 14.8	130.4 ± 14.0	0.118
DBP(mmHg)	70.8 ± 11.9	69.2 ± 11.6	0.322	71.7 ± 10.8	70.5 ± 9.7	0.199
Pulse rate (/min)	75.3 ± 12.3	74.7 ± 11.6	0.492	73.6 ± 11.8	73.3 ± 11.5	0.604

SBP, systolic blood pressure; DBP, diastolic blood pressure.

**Table 6:** Changes in hemodynamics before and after mirabegron treatment classified by antihypertensive drug.

	SBP			DBP			PR		
	0 W	12 W	*p*	0 W	12 W	*p*	0 W	12 W	*p*
ARBs	131.4 ± 15.4	130.5 ± 14.7	0.216	69.9 ± 10.8	70.5 ± 9.9	0546	74.0 ± 12.3	74.6 ± 9.4	0.931
CCBs	132.4 ± 12.5	131.3 ± 18.2	0.203	73.5 ± 11.8	70.5 ± 10.1	0.111	73.8 ± 12.6	73.1 ± 15.9	0.662
ARBs + CCBs	133.1 ± 16.3	130.6 ± 15.7	0.571	71.6 ± 11.9	70.7 ± 9.2	0.571	72.8 ± 12.4	72.0 ± 9.8	0.377
ARB + diuretics	130.4 ± 16.6	132.6 ± 14.2	0.734	71.4 ± 7.1	70.3 ± 9.7	0.613	75.3 ± 10.4	73.8 ± 10.6	0.270
Diuretics	140.4 ± 8.8	135.0 ± 17.1	0.625	78.2 ± 7.5	69.4 ± 13.4	0.250	68.2 ± 3.7	68.0 ± 5.7	0.750

SBP, systolic blood pressure; DBP, diastolic blood pressure; ARBs, angiotensin II receptor blockers; CCBs, calcium channel blockers; BPH, benign prostatic hyperplasia.

**Table 7:** Univariate analysis for the risk of systolic blood pressure elevation.

	Univariate Analysis		
	OR	95% CI	*p*
Age (≥65 years)	2.77	1.13–8.31	0.024
Gender: male	0.96	0.53–1.76	0.902
Body mass index	0.97	0.90–1.05	0.394
Hypertension: presence	0.77	0.41–1.40	0.389
Diabetes Miletus: presence	1.04	0.37–2.55	0.939
Hyperlipidemia: presence	1.14	0.48–2.47	0.757
Chronic renal dysfunction: presence	1.03	0.52–1.96	0.925

OR, odds ratio; CI, confidence interval.

**Table 8:** Baseline characteristic data of SBP elevation after propensity score matching.

	<65 y. o (Young Group) *n* = 51	≥65 y. o (Elderly Group) *n* = 51	*p*	Standardized Mean Differences
Gender (male/female)	15/36	15/36	0.786	0.089
Body mass index (kg/m^2^)	23.1 ± 5.2	22.4 ± 4.2	0.481	0.028
Hypertension (%)	12 (23.5)	13 (25.5)	1.000	0.075
Diabetes Miletus (%)	6 (11.8)	5 (9.8)	1.000	0.099
Hyperlipidemia (%)	3 (5.9)	1 (2.0)	0.617	0.098
Chronic renal dysfunction (%)	9 (17.6)	7 (13.7)	0.786	0.065
SBP at baseline (mmHg)	123.8 ± 15.2	121.6 ± 12.8	0.411	0.001

SBP; systolic blood pressure.

**Table 9:** Difference in the clinically significant elevation of SBP between ages.

	<65 Years (Young Group) *n* = 51	≥65 Years (Elderly Group) *n* = 51	*p*
SBP elevation (%)	4 (7.8)	18 (35.3)	0.002

SBP; systolic blood pressure.

## Data Availability

The data presented in this study are available on request from the corresponding author. The data are not publicly available due to privacy/ethical restrictions.
